# Current concepts in imaging and endovascular treatment of acute ischemic stroke: implications for the clinician

**DOI:** 10.1186/s13244-019-0744-4

**Published:** 2019-06-13

**Authors:** Thijs van der Zijden, Annelies Mondelaers, Laetitia Yperzeele, Maurits Voormolen, Paul M. Parizel

**Affiliations:** 1 Department of Radiology, Antwerp University Hospital & University of Antwerp, Wilrijkstraat 10, 2650 Edegem, Belgium; 2 Department of Neurology, Antwerp University Hospital & University of Antwerp, Wilrijkstraat 10, 2650 Edegem, Belgium; 3 Department Translational Neurosciences, University of Antwerp, Universiteitsplein 1, 2610 Wilrijk, Belgium; 4 Faculty of Medicine and Health Sciences, University of Antwerp, Universiteitsplein 1, 2610 Wilrijk, Belgium

**Keywords:** Acute stroke, Patient selection, Endovascular procedure, Cerebrovascular accident, Diagnostic imaging

## Abstract

During the last decade, the management of acute ischemic stroke has changed dramatically, from an expectant bedside “wait and see” attitude towards active treatment, thanks to the continuous improvement of new therapeutic options. In addition to the use of intravenous (IV) thrombolysis in emergent large vessel occlusion (ELVO), endovascular therapy (EVT) has proven to be very efficient in selected acute stroke patients. The indications for EVT have progressed from the era of thrombolysis to individual patient profiling. Recently, several indication parameters, e.g., “treatment time window” or “more distal vessel occlusion,” are under debate for adjustment. In this article, we review the imaging strategies in acute stroke and discuss several EVT indication dogmas, which are subject to change.

## Key points

- Endovascular recanalization treatment has become the predominant therapy of acute ischemic stroke due to large vessel occlusion.
- Neuroimaging, including basic vessel-imaging, is mandatory for proper patient selection for endovascular therapy (EVT).
- The use of advanced neuroimaging techniques (CT or MRI perfusion and/or DWI MRI) allows selection of patient with late-presenting (> 6 h) or wake-up stroke with large vessel occlusion for EVT.
- Indication setting for EVT in acute stroke is not fixed and is subject to changes in clinical insights and development of current and new recanalization techniques and devices.

## Introduction

**Fig. 1:**
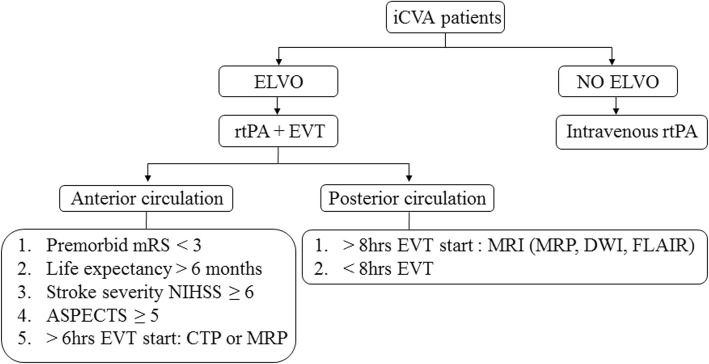
Schematic overview of EVT selection guidelines used for anterior circulation stroke patients

Ischemic stroke is an ever-growing and highly significant worldwide health problem. The incidence of acute ischemic stroke in the USA is about 800,000 patients per year (0.25% of the total population), and this number is expected to increase significantly in the next decades, due to the aging population [1]. Stroke does not only directly impact patients, but also their immediate environment and society as a whole. Approximately one third of stroke patients remain completely or partially dependent on their caregivers [2]. Before the start of regular clinical implementation of intravenous thrombolysis in the late 1990s, no primary acute stroke treatment tool was available. The development of thrombectomy medical devices followed shortly after the beginning of the thrombolysis era. After the introduction in acute stroke treatment of detachable stents, specifically designed as an adjunctive tool in cerebral aneurysm treatment [3], the concept of using a “stent retriever” as an endovascular thrombectomy device was embraced by the interventional neuroradiology community. However, after the initial enthusiasm, the increasing use of stent retrievers was halted due to the publication of three randomized controlled trials (RCTs) in 2013, reporting the non-superiority of endovascular treatment versus intravenous treatment alone [4–6]. At the beginning of 2015, the paradigm of acute stroke treatment shifted ultimately back to endovascular treatment. Several RCTs clearly confirmed the benefits of using endovascular thrombectomy devices on the clinical outcome of stroke patients, compared to those receiving only standard medical care [7–9]. The past years, a plethora of recommendations for endovascular treatment (EVT) have been issued with ongoing revisions. In Europe, the consensus European Stroke Organization (ESO)-Karolinska Stroke Update statements are published and revised regularly in collaboration with the European Society of Neuroradiology (ESNR) and the European Society for Minimally Invasive Neurological Therapy (ESMINT) [10]. On January 24, 2018, a revised version of the American Heart Association (AHA)/American Stroke Association (ASA) medical guidelines, based on available Class I and Class IIa scientific evidence, was published [11]. In practice, the implemented stroke treatment guidelines in individual hospitals are also influenced by more pragmatic factors, such as the local healthcare organization and infrastructure, local stroke treatment expertise, and availability of stroke specialists. These protocols list the proper indications for mechanical thrombectomy in certain circumstances, regarding items such as the premorbid status of the patient, time interval between onset of stroke symptoms and intended treatment, and appreciation of core infarct size versus potentially salvageable brain tissue. For instance, the Antwerp University Hospital follows the guidelines, which are schematically represented in Fig. 1. Because of the progress in endovascular device technology and new clinical insights, the focus of clinical research is lately evolving from the classical indications, originating from the era of intravenous and intra-arterial thrombolysis to more atypical indications. These indications allow expansion of the window of treatment opportunities. Some of the issues dealt with include acute stroke treatment of very elderly patients (> 85 years of age), recanalization treatment effect in patients with relative unfavorable premorbid clinical condition (modified Rankin Scale (mRS) > 2) [12], patients presenting beyond the time window of 6 h, patients with unknown time of onset (including wake-up stroke), patients with low Alberta Stroke Program Early CT scores (ASPECTS) or poor collateral status, treatment of tandem occlusions, posterior circulation strokes, or more distal vessel occlusions (distal M2, proximal M3 occlusions), and the use of bridging thrombolysis (intravenous thrombolysis plus EVT) therapy in emergent large vessel occlusion (ELVO) patients if EVT is not immediately available. In this article, we present state-of-the-art practice in (neuro-) radiological management of acute stroke patients. We review the necessary neuroimaging steps, attitude towards primary treatment time window and EVT indication setting, and EVT in more uncommon situations as listed above.

## The growing role of imaging and image-guided treatment in the management of acute stroke patients

**Fig. 2:**
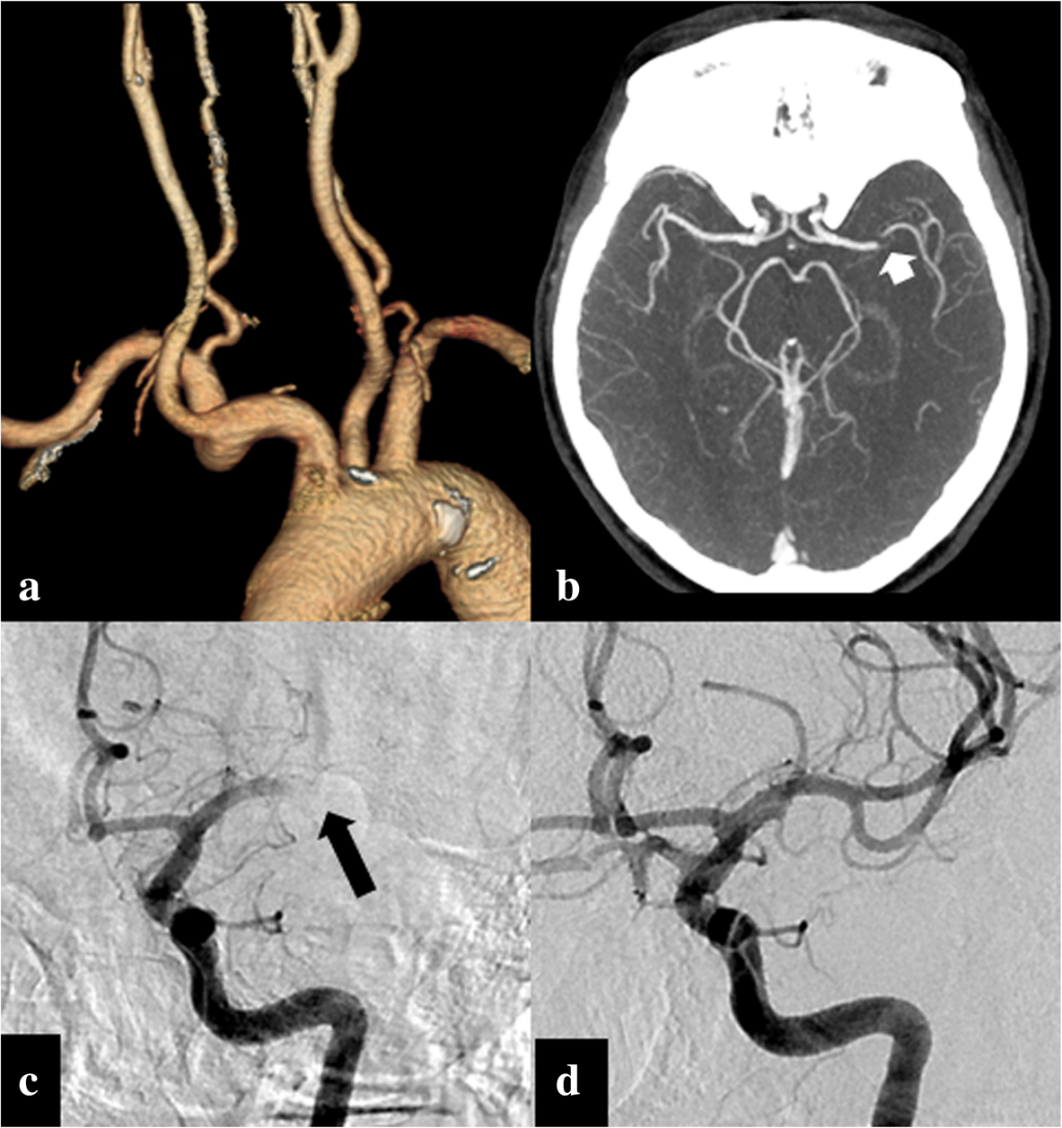
This 78-year old woman with atrial fibrillation presented with sudden onset of right-sided hemiplegia and aphasia. CT-angiography (CTA) with 3D volume rendering shows normal patency with moderate tortuosity of the cervical carotid arteries (**a**). An axial maximum intensity projection (MIP) reformation of the CTA demonstrates an occlusion in the M1 segment of the left MCA (**b**, white arrow). The patient was referred immediately for EVT. Catheter digital subtraction angiography, in anteroposterior (AP) projection, confirmed the left MCA M1 segment occlusion (**c**, black arrow). Subsequently, a successful endovascular removal of the thrombus was performed (**d**, post EVT AP projection angiography). At clinical follow-up, a complete resolution of the neurological symptoms occurred soon after EVT

In order to select for patients eligible for EVT, adequate imaging is mandatory. This implies that imaging protocols should be practical and fast, both in the availability of equipment and quickness of image acquisition. In most cases, a “one-stop-shop” approach relies on computed tomography (CT), because of its widespread availability, short image acquisition times, the possibility to monitor the patient in the machine, and the rapid subsequent interpretation of the images. The first step is to perform a non-contrast CT of the brain to rule out hemorrhage, and/or the presence of stroke mimickers, or signs of a significant core infarct. The next step should be the confirmation of the presence of a large vessel occlusion, which is mostly done by CT angiography [13]. In patients with severely compromised kidney function, magnetic resonance angiography (MRA) 3D time-of-flight (TOF) or conventional angiography can be considered. It is important to visualize not only the cranial arteries, but also the cervical arteries including the aortic arch, given the fact that an additional vascular lesion at the cervical level is observed in up to 20% of cases [14]. Figure 2 illustrates the use of CT angiography for both confirmation of an intracranial culprit lesion and for mapping of the craniocervical vascular anatomy when considering a catheter-based procedure.

**Fig. 3:**
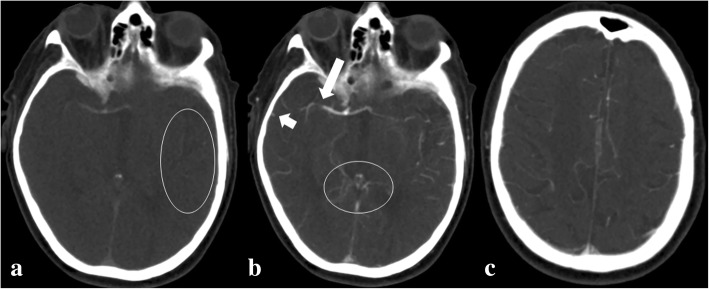
A 73-year old woman presented with sudden onset of left hemiplegia. In addition to non-contrast CT brain and CT angiography of the craniocervical arteries, CT perfusion was performed because of the uncertainty of time of symptom onset. The presented source images are images at consecutive time intervals. Source image at early arterial phase (**a**) shows discrete contrast filling into temporal M2 and proximal M3 branches at the normal left side (encircled). At the right side, there is only filling of the proximal middle cerebral artery with slight contrast penetration past the thrombus, but no contrast at the level of more distal M2 segment or proximal M3 segments. Source image in the later arterial phase (**b**, 4.5 s after **a**) shows almost symmetrical filling of distal arterial branches of both hemispheres. The short occlusion at distal M1 segment (long arrow) is better appreciated. The central cerebral veins are symmetrically opacified (encircled). There is already filling of the right superficial middle cerebral vein (short arrow), but persisting asymmetry in opacification of the cortical veins is observed. In late venous phase (**c**, 6 s after **a**), source image centered at a more cranial level shows symmetrical cortical vein opacification. Because of deemed adequate collateral vascular status and large penumbra/infarct mismatch at perfusion imaging (not shown), subsequently, the patient underwent successful retrieval of a small thrombus. Almost immediately after the procedure, a good clinical recovery occurred with uneventful post EVT clinical course

We consider that the previously mentioned imaging steps constitute the minimal requirements for proper EVT. More advanced neuroimaging techniques might be beneficial to assess the potential success of an EVT procedure. Perfusion imaging can provide a practical estimate of the size of the core infarct versus the amount of potentially salvageable brain [15, 16]. MRI is able to ascertain a mismatch between infarcted brain areas versus penumbra and/or oligemia areas, using DWI, fluid-attenuated inversion recovery (FLAIR) imaging, and MR perfusion [17]. Since a couple of years, the assessment of pre-treatment collateral status of individual patients with imaging has become popular, as it can be associated with the rates of successful revascularization in acute ischemic stroke patients who undergo EVT [18]. The collateral status in a given patient refers to the presence or absence of collateral vascular networks able to stabilize or maintain cerebral blood flow (CBF) in case of occlusion of the main arterial supply. This network can consist of extra-to intracranial anastomoses, interconnections at the level of the circle of Willis, leptomeningeal collaterals, or medullary collaterals [19]. Collaterals may vary considerably in size, number, and location among individuals [20]. The efficiency of a collateral vascular network is influenced by patient age, the presence of metabolic syndrome and hyperuricemia, mean arterial blood pressure, and cerebral blood perfusion [21–23]. This may imply that in some ELVO individuals, large core infarcts can develop within minutes, whereas in other ELVO patients, thanks to a good collateral vascular network, a potentially reversible oligemic state or penumbra can exist for several hours before turning into a core infarct [24]. The confirmation of the presence or absence of a collateral network can become a promising potential biomarker for prediction of clinical outcome in acute ischemic stroke in case endovascular treatment is considered. Even though conventional angiography is still the gold standard technique for collateral status assessment, CT angiography can be valuable for appraisal of contrast filling of vessels in the brain area at risk for infection. A more profound representation of the collateral vascular status in ELVO patients can be made by using multiphasic CT angiography or CT perfusion source images by adding a dynamic, time component compared to conventional monophasic CT angiography, which can only provide a snap-shot in time [25]. Figure 3 shows us how CT perfusion source images can be used for assessment of the collateral vascular status. Table 1 suggests an advanced CT protocol for stroke including pre-contrast CT scan, multi-phase CT angiography, and perfusion imaging.

**Table 1:** A proposed advanced CT imaging protocol for selecting acute ischemic stroke patients for EVT

Imaging steps	Technical notes	Questions to be answered
Non-contrast CT	• Parenchymal brain imaging	• Hemorrhage? Alternative diagnosis? • ASPECTS
Head and neck CT angiography	• From aortic arch to vertex • 60–80 mL, 350 mg iodide/mL	• Small or large vessel occlusion? • Tandem lesion? • Roadmap for EVT
Brain 4D CT angiography	• Low dose • Multiple scans	• Collateral vascular status?
Brain perfusion	• Brain coverage, depending on available scan width • 40–60 mL, 350 mg iodide/mL	• Infarct volume • Mismatch penumbra/infarct

The starting point of acute stroke patient evaluation always consists of non-enhanced imaging of the brain. The way of implementation of the following imaging steps can be tailored to the specific clinical situation (e.g., time window after symptom onset, clinical stroke syndrome).

## What about the time window for endovascular treatment (EVT)?

**Fig. 4:**
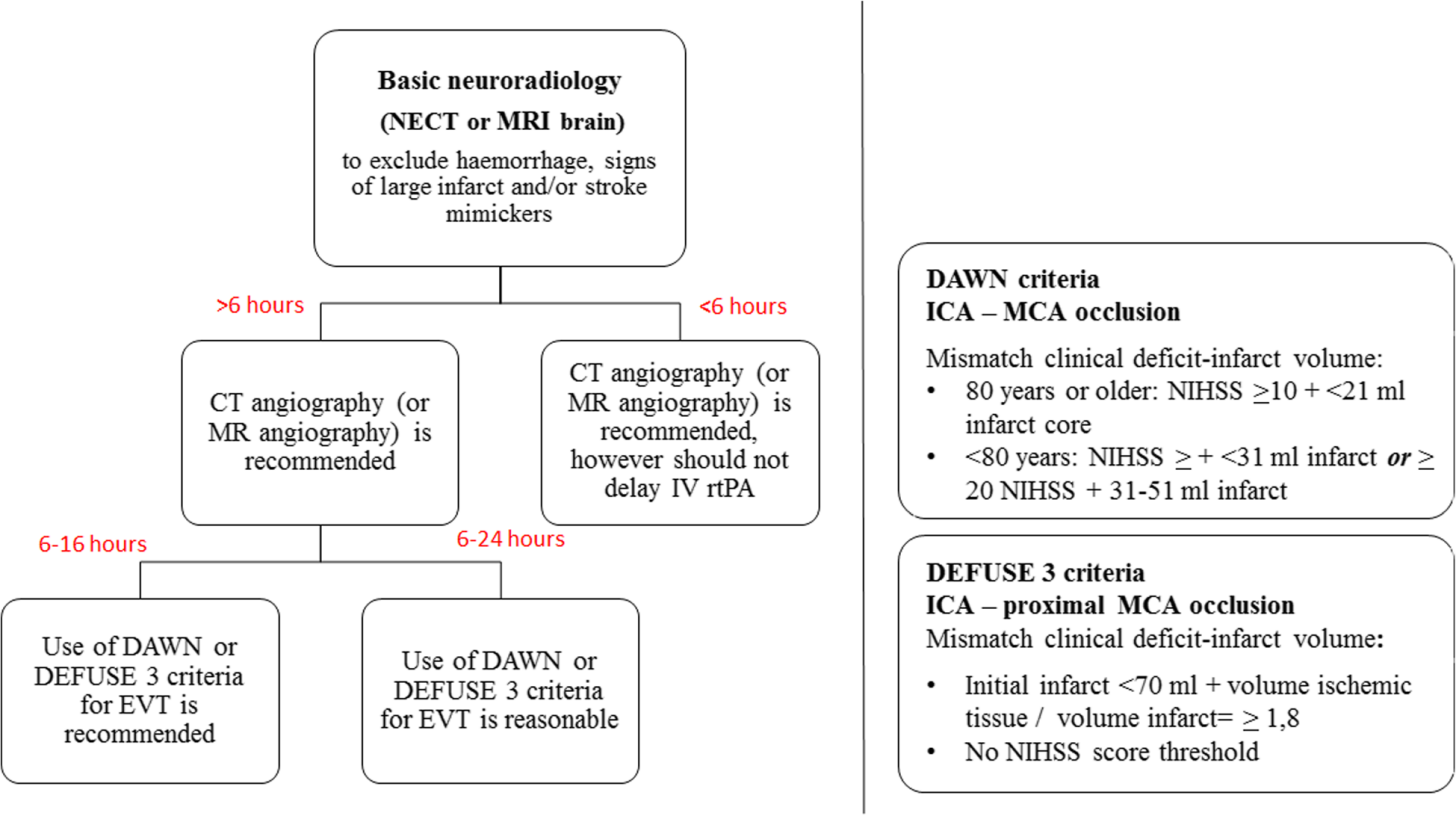
Flowchart representing patients’ selection criteria for EVT within 6 h and beyond the 6-h time window according to class I and IIA recommendations

**Fig. 5:**
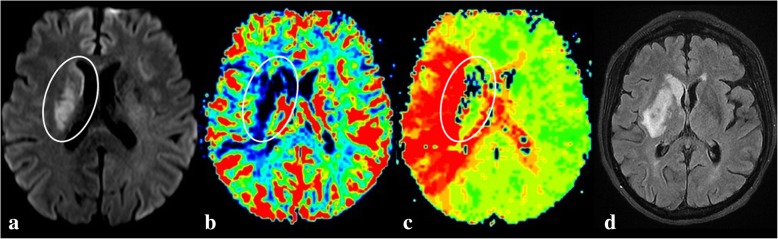
A 67-year woman was admitted with a wake-up stroke. Clinically, she presented with severe right MCA infarct symptoms (NIHSS 14). Since the time of onset of the stroke could not be determined, MRI was performed. The diffusion-weighted trace image (**a**) shows an infarct core with diffusion restriction in the right basal ganglia. The parametric MR perfusion maps (**b**, cerebral blood volume (CBV) and **c**, time to peak (TTP)) show a mismatch indicating a large penumbra, i.e., area of potentially salvageable brain tissue. Subsequently, a successful mechanical thrombectomy was performed and the patient had a substantial recovery (NIHSS 3). Follow-up MRI on day 3 after treatment (**d**) shows that the core infarct remains limited within the right basal ganglia with sparing of the insular, frontal, and parietal cortex

**Fig. 6:**
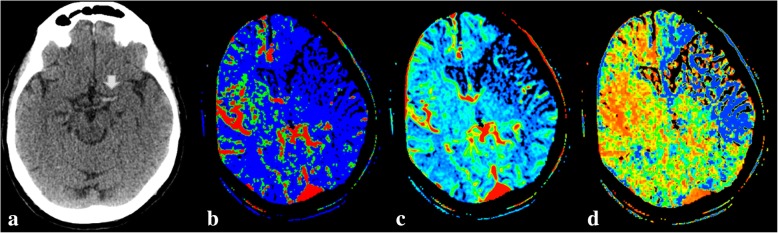
This 75-year-old woman presented with right hemiplegia and aphasia. Axial unenhanced CT shows a hyperdense vessel sign in the left MCA M1 segment (**a**, white arrow). CT perfusion was also performed, with parametric maps indicating CBF (**b**), CBV (**c**), and mean transit time (MTT) (**d**). These images reveal a large established core infarct with no significant penumbra. This indicates a complete stroke; no endovascular treatment was attempted

Until recently, treating patients beyond the 6 h time window after the onset of stroke symptoms was considered off-label. Also, patients presenting with wake-up strokes were not considered as proper EVT candidates. The use of a 6-h time window in acute stroke treatment was based on data concerning intra-arterial thrombolysis [26, 27]. This time window was considered to represent the best compromise between potential treatment benefit and relative risk of complications. It seems that the use of advanced neuroimaging criteria alone for the selection of potential non-responders to EVT within the 6-h time window will not modify the treatment effect [28, 29]. The strict application of a time window entails the risk of excluding patients with residual large penumbra at 6 h after onset (so-called slow stroke progressors) [24]. A recent publication of the DAWN (DWI or CTP Assessment with Clinical Mismatch in the Triage of Wake-up and Late-Presenting Stroke) trial indicated that the time paradigm shifts to a more individual patient-specific timeline, based on more advanced neuroimaging methods [30]. The DAWN trial was set up as a multicentric (26 centers) RCT, in which late-presenting acute stroke or wake-up stroke patients with terminal carotid and/or M1 segment middle cerebral artery occlusions were randomized into a group treated with both EVT and best medical care versus a control group receiving only best medical care. Inclusion criteria included a treatment time window from 6 to 24 h after last seen well and a clear mismatch between infarct size on imaging and clinical symptoms based on the National Institutes of Health Stroke Scale (NIHSS) score. Core infarct volumes were computed by using automated software (RAPID, iSchemaView). The primary endpoints were the mean value for disability utility weighted mRS score and the proportion of patients with a mRS of 0–2 at 90 days clinical follow-up. The trial was prematurely halted because of the clear benefit of EVT. Functional independence was 49% in the EVT arm of 105 patients versus only 13% in the best medical therapy group of 102 patients. Rates for death and significant intracranial hemorrhage were statistically not significant between both groups [30]. The potential positive effect of EVT on clinical outcome of late-presenting acute stroke patients is also confirmed in other trials, such as the DEFUSE 3 trial [31]. In the DEFUSE 3 trial, ELVO patients were selected using advanced neuroimaging as well, but the applied time window after last seen well was 6–16 h instead of 6–24 h in the DAWN trial. In contrast to the DAWN trial, the DEFUSE 3 used a universal infarct volume cutoff (70 mL compared to an age- and NIHSS-dependent cutoff) [30, 31]. Figure 4 represents patients’ selection criteria for EVT within 6 h and beyond the 6 h time window according to the class I and IIA recommendations [11]. Although ideally primary acute stroke treatment should be carried out as soon as possible, it now appears feasible to select late-presenting stroke patients for EVT by using advanced imaging tools. In our practice, the positive results of these trials have changed our treatment policy regarding late-presenting acute stroke patients. We perform additional CT perfusion imaging and/or MRI with DWI, FLAIR, and/or MR perfusion imaging in all acute stroke patients presenting outside of the classic treatment time window, as illustrated in Figs. 5 and 6.

## Less common clinical situations in endovascular treatment (EVT)

The ideal situation for EVT is represented by patients with a single occlusive lesion arriving at the hospital within the shortest possible time, with non-hemorrhagic stroke and high NIHSS. Unfortunately, the clinical reality is oftentimes different. In the next paragraphs, we will discuss some “less common” and also “suboptimal” situations.

## Tandem lesions

Tandem lesions are defined as (sub) occlusive “double” lesions, located both proximally (at the level of the cervical internal carotid artery) and distally (at the level of the intracranial arteries). In 10–20% of acute strokes, tandem lesions are present [14]. The most common causes of cervical lesions are dissection (especially in younger people) and atherosclerotic plaques [32]. Contrary to popular belief, a cervical carotid artery occlusion is not a contra-indication for EVT. Although not always successful, in practice, it is often surprisingly easy to navigate a distal access catheter through the proximal lesion for treatment of the distal lesion. The primary focus for treatment should be the symptomatic lesion (the so-called culprit lesion), which is almost invariably the distal lesion. There is an ongoing debate among interventional neuroradiologists whether or not to treat the proximal lesion first in order to get proper access to the distal lesion [33, 34]. Stenting of the cervical lesion in the acute phase can be useful in order to prevent recurrent stroke [35], especially in atherosclerotic disease. We are not aware of any large patient series concerning the safety and effectiveness of stenting of tandem lesions in the acute phase of stroke. In one publication of the RECOST study, it was stated that a conservative approach (without stenting) in case of adequate primary collateral circulation by the circle of Willis may be reliable and safe [36]. It is important to note that early treatment with anti-aggregants, after intravenous thrombolysis, might increase the risk of (symptomatic) hemorrhagic transformation during or after stenting of stroke patients [37]. A potential complication after treatment of a chronic cervical carotid artery subocclusion is the occurrence of cerebral hyperperfusion syndrome [38]. It is believed that this syndrome is caused by impairment of the cerebrovascular autoregulation. Clinical symptoms include headache, seizures, and focal neurological symptoms. If not adequately detected and treated, brain edema and hemorrhage can develop, potentially leading to death [39]. In our practice, we limit carotid stenting and/or balloon angioplasty to patients with persisting hemodynamic insufficiency in the acute phase.

## Patients with low NIHSS

A significant proportion of patients with large vessel occlusions present initially in relatively good clinical condition, i.e., with a low NIHSS. However, the natural course of the disease can lead to a secondary worsening of clinical symptoms. In these situations, the neurovascular team is confronted with a dilemma, because, in case of persisting occlusion, the risk of secondary clinical worsening in the acute or subacute phase increases [40, 41]. A recent, retrospective multicenter cohort study, which included 214 patients, showed no improvement in independent functional outcomes in mild strokes (NIHSS < 6) in patients receiving EVT compared to medical treatment, irrespective of occlusion location, with increased symptomatic intracerebral hemorrhage rates [42]. Therefore, it needs to be determined if it is justifiable to expose patients with modest symptomatology to the potential risks of a recanalization procedure. Furthermore, the occurrence of spontaneous recanalization of large vessel occlusions has been described [43]. Two other studies, however, demonstrated the effectiveness of mechanical thrombectomy with excellent clinical outcome for patients with low NIHSS score (< 5) undergoing endovascular recanalization for large vessel occlusion in the anterior circulation [44, 45]. Until now, there are no clear guidelines on EVT in ELVO patients with low NIHSS. In such cases, it is advisable to assess the collateral vascular status of the patient [46]. The risk of treatment should always be carefully weighed against the expected potential benefits, in order to make the best possible decision for the individual patient.

## Endovascular treatment in distal occlusions

The benefit of EVT in acute ischemic stroke patients was established in RCTs for proximal large vessel occlusions, focusing exclusively on the internal carotid artery and proximal middle cerebral artery (M1 segment) [7–9, 47]. However, the effectiveness of mechanical thrombectomy in more distally located lesions, e.g., at the level of distal M2 segment or proximal M3 segment of the middle cerebral artery, has not been determined yet. After intravenous (IV) thrombolytic therapy alone, patients with distal lesions tend to show a more favorable clinical outcome when compared to patients with more proximally located lesions [48]. Since IV thrombolysis has proven to be more efficient in smaller clots [49], IV treatment might offer a relative advantage over EVT for smaller vessel occlusions. In practice, EVT at the level of more distally located smaller vessels with thinner vessel walls can be technically more challenging, with potentially a higher risk of complications. Nevertheless, it is proven that EVT of more distal occlusions, i.e., isolated M2 occlusions, is just as safe and effective as EVT performed in proximal large vessel occlusions [50, 51]. Therefore, in specific cases presenting with a significant neurological deficit due to a strategic small vessel occlusion (e.g., patients with aphasia as a single symptom), EVT should be considered as a valuable treatment tool. In such cases, the decision whether or not to treat the patient with EVT should be made based on symptoms, age, and clinical status of the patient, pre-existent vascular disease, and the precise location of the distal vessel occlusion.

## Acute basilar artery occlusion

**Fig. 7:**
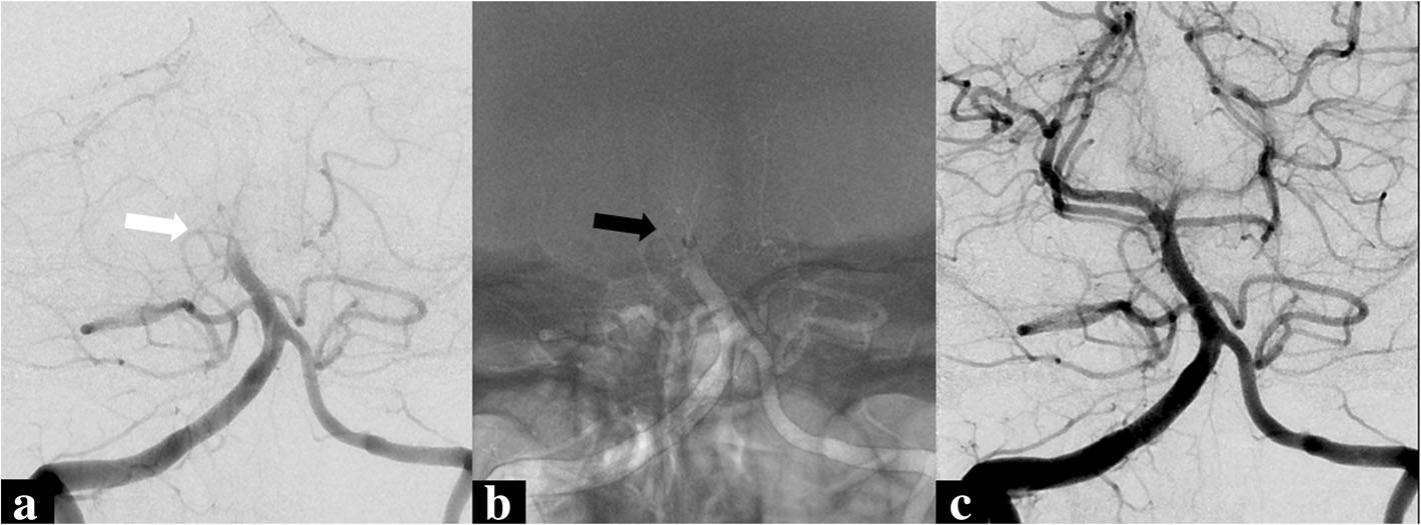
A 72-year-old woman with a left hemi-paralysis, dysarthria, and anisocoria (NIHSS 22). She was treated initially with IV thrombolysis but did not recover and was referred to our department for EVT. Catheter angiography, AP projection, with contrast injection in the dominant right vertebral artery reveals a midlevel occlusion of the basilar artery (**a**, white arrow). Thrombus removal by means of an aspiration catheter was attempted as illustrated by a fluoroscopy image with vessel road map projection (**b**, with the black arrow at the level of the catheter tip at the proximal end of the thrombus). After thrombus aspiration, an AP projection angiogram showed successful recanalization with patency of the basilar artery (**c**)

Approximately 20% of all ischemic strokes occur in the posterior circulation; this group includes patients with basilar artery occlusion (BAO), who have a very poor prognosis. For instance, in the prospective Basilar Artery International Cooperation Registry Study (BASICS), 402 (68%) out of the 592 analyzed patients had a poor clinical outcome, defined as a mRS of 4 or 5 or death at 1 month [52]. The superiority of EVT over IV thrombolysis (IVT) was not supported in this study, and so the authors suggested to set up RCTs to evaluate the efficacy of EVT versus IVT only in patients with an acute BAO. Although mechanical thrombectomy in the basilar artery can be performed quite successfully (Fig. 7), EVT in BAO has not yet been studied in large RCTs. The follow-up of the BASICS registry, so-called BASICS trial, was designed as a multicenter open-label phase III interventional RCT, assessing the efficacy and safety of EVT in patients with BAO [53]. Patients were randomized between additional EVT followed by optimal medical care versus optimal medical care alone. However, after inclusion of the first patient in October 2011, patient recruitment has been exceeding slow and the trial is still ongoing. It is not unthinkable that many patients, who should have been included in the study, are simply treated by EVT on an individual basis. It seems likely that patient selection is therefore skewed towards patients with a more doubtful prognosis; this can create a significant study bias resulting in a less favorable study outcome regarding EVT in BAO.

The current guidelines indicate that the use of mechanical thrombectomy with stent retrievers is considered as “reasonable” to treat carefully selected patients who have “causative occlusion of the basilar artery” [10, 11]. In our practice, advanced imaging with DWI plays an important role in selecting candidates with vertebrobasilar artery occlusion for EVT by providing information about the extent and location of the infarcted areas.

## Conclusion

When dealing with ELVO patients who might be eligible for EVT, knowledge about the occlusion site and infarct volume are of paramount importance. Adequate and fast pre-endovascular stroke treatment vessel imaging is vital in selecting suitable patients. A good impression of the potential benefit of EVT can be achieved by the assessment of collateral vascular networks in ELVO patients, and this can be done in a robust and fast way by the use of (multiphasic) CT angiography. In more doubtful cases, it is advisable to apply more advanced neuroimaging techniques, such as CT perfusion or MR DWI and/or perfusion imaging. The policy concerning indication setting for EVT in ELVO is rapidly changing; treatment paradigms, e.g., time windows and sites of occlusion, are constantly being questioned and adjusted, based on evidence from RCTs. In addition to the clinical decision-making process, patient profiling on the basis of advanced neuroimaging is becoming increasingly important. Individual assessment is taking precedence over patient group profiling, and this highlights the increasing role of the neuroradiologist in the management of acute stroke patients.

## Abbreviations

AHA/ASA: American Heart Association/American Stroke Association
ASPECTS: Alberta Stroke Program Early CT Score
BAO: Basilar artery occlusion
BASICS: Basilar Artery International Cooperation registry Study
CBF: Cerebral blood flow
CBV: Cerebral blood volume
ELVO: Emergent large vessel occlusion
ESMINT: European Society for Minimally Invasive Neurological Therapy
ESNR: European Society of Neuroradiology
ESO: European Stroke Organization
EVT: Endovascular therapy
FLAIR: Fluid-attenuated inversion recovery
ICA: Internal carotid artery
IV: Intravenous
MCA: Middle cerebral artery
MERCI: Mechanical Embolus Removal in Cerebral Ischemia
MIP: Maximum intensity projection
mRS: modified Rankin Scale
MTT: Mean transit time
NIHSS: National Institutes of Health Stroke Scale
RCTs: Randomized controlled trials
rtPA: recombinant tissue Plasminogen Activator
TTP: Time to peak
